# A non-parametric method for determining epidemiological reproduction numbers

**DOI:** 10.1007/s00285-021-01590-6

**Published:** 2021-03-15

**Authors:** Frank P. Pijpers

**Affiliations:** Statistics Netherlands & Korteweg-de Vries Institute for Mathematics, University of Amsterdam, Amsterdam, Netherlands

**Keywords:** Transmission, Infectious diseases, Reproduction number, Estimation techniques, Covid-19, 45, 92

## Abstract

In the spreading of infectious diseases, an important number to determine is how many other people will be infected on average by anyone who has become infected themselves. This is known as the reproduction number. This paper describes a non-parametric inverse method for extracting the full transfer function of infection, of which the reproduction number is the integral. The method is demonstrated by applying it to the timeline of hospitalisation admissions for covid-19 in the Netherlands up to May 20 2020, which is publicly available from the site of the Dutch National Institute of Public Health and the Environment (rivm.nl).

## Introduction

The reproduction rate is a fundamental concept in epidemiology. One quantifier of this is the basic reproduction number $R_0$, which is the average number of secondary cases generated by a typical infectious individual over the entire course of the infectious period in a fully susceptible population. The other is the generation time: the time interval between the infection time of the infector and her/his infectees. These definitions are reproduced from Liu et al. (2018) which itself also refers to the book of Anderson and May (1991) and papers of Roberts and Heesterbeek (2007) and Wallinga and Lipsitch (2007).

In the paper of Nishiura (2010), which bases itself on the paper of Diekmann et al. (1990), the following basic equation is presented. Let *j*(*t*) be the number of new infections (i.e., incidence) at calendar time *t*. Supposing that each infected individual on average generates secondary cases at a rate $A(\tau )$ at time $\tau$ since infection (where $\tau$ is referred to as the “infection-age” or time delay hereafter), *j*(*t*) is written as:

1 $$\begin{aligned} j(t)= \int \limits _{0}^{\infty } A(\tau ) j(t-\tau )\mathrm{d}\tau \end{aligned}$$

where the integral expresses a convolution of $A(\tau )$ and *j*(*t*). It is important to mention that Eq. (1) is in fact a more restricted form than what is presented in Diekmann et al. (1990). The more general linearized real time form for a heterogeneous population, with a total number per group in the population *S*, and a transmission function $A_g$, is:

2 $$\begin{aligned} j_g(t, \xi )=S(t,\xi ) \int \int \limits _{0}^{\infty } A_g(t,\tau , \xi ,\eta ) j_g(t-\tau , \eta )\mathrm{d}\tau \mathrm{d}\eta \end{aligned}$$

where the variable $\xi$, and its counterpart under the integral sign $\eta$, are used to express explicitly that the population is heterogeneous, i.e. is subdivided in groups. To which group an individual in the population belongs is briefly referred to as their state. There can be heterogeneity in many different ways, and wherever this implies discrete groups, the integral over $\eta$ becomes a summation over the discrete values it can take. In the present context it is necessary to distinguish at least three groups:

- Those in the population that are still susceptible, and not infectious
- Those in the population that are infectious
- Those who no longer are. These have either gained immunity after they have recovered from infection, or they are (progressively) withdrawn from the population, for instance because effective measures are in place to completely isolate them from the rest.

The function *S* and also $A_g$ must be functions of *t* as well, which is not explicitly mentioned in Diekmann et al. (1990). One reason for this is that as time progresses, even in the absence of any isolation measures, the third group will increase in size through recovery and immunity and hence the other two groups must decrease. In Eq. (2) the normalization is chosen such that $S(0, \xi )/N_{pop}$ is the density function of that portion of the population, of size $N_{pop}$, that is susceptible at times prior to the disease being introduced. In Eq. (2) the $j(t, \xi )$ is the rate at which susceptibles with state $\xi$ are infected at time *t*. If now the relationship between *j* and $j_g$, is written as:

3 $$\begin{aligned} j(t)\equiv \int j_g(t, \xi )\mathrm{d}\xi \end{aligned}$$

and in addition it is assumed that $A_g$ satisfies:

4 $$\begin{aligned} \int S(t,\xi ) A_g(t, \tau , \xi ,\eta )\mathrm{d}\xi = A(t, \tau ) \end{aligned}$$

then integrating Eq. (2) over $\xi$, and interchanging integrations produces the form Eq. (5), except that now *A* still has an explicit time dependence. Since $R_0$ represents the total number of secondary cases that a primary case generates during the entire course of infection, starting from a fully susceptible population, it is the integral of $A_g$ at $t=0$:

5 $$\begin{aligned} R_0= \iint \ \int \limits _{0}^{\infty } A_g(0, \tau , \xi , \eta ) \mathrm{d}\tau \ \mathrm{d}\xi \mathrm{d}\eta \end{aligned}$$

In a finite population, the fraction of the population that has been infected is a reservoir which can re-infect others over some period of time, governed in part by the virology, i.e. infectiousness, and in part by whether they isolate themselves or are isolated because their symptoms are sufficiently clear to indicate the need for such measures. An effective reproduction number, taking all this into account, therefore cannot be a constant but must instead still be a function of time:

6 $$\begin{aligned} R(t)= & {} \iint \ \int \limits _{0}^{\infty } S(t,\xi ) A_g(t, \tau , \xi , \eta ) \mathrm{d}\tau \ \mathrm{d}\xi \mathrm{d}\eta \nonumber \\= & {} \int \limits _{0}^{\infty } A(t,\tau ) \mathrm{d}\tau \end{aligned}$$

Eq. (4) might appear quite restrictive, since the dependence on $\eta$ on the left hand side must disappear. However, if the options for $\eta$ are “susceptible”, “infectious” or “removed”, then $A_g$ can be non-zero only for the second of these categories, so that the dependence on $\eta$ drops out of the equation.

The explicit dependence of *A* on *t* as well as $\tau$ is more problematic. In early stages of an epidemic, without effective isolation measures, and with the “susceptible” group of the population only changing negligibly because the “removed” group is still extremely small, it could be argued that time-independence for *S* and $A_g$ is a good approximation. In what follows an additional approximation is used, which is equivalent to allowing separation of time scales where the time evolution of *A* is slow compared to its variation with $\tau$. One way to express the notion that the derivative of *A* with respect to *t* is always small compared to the derivative of *A* with $\tau$ is to write a formal expansion in terms of a small parameter $\epsilon$:

7 $$\begin{aligned} A(t,\tau )= & {} A_0(\tau ) + \epsilon A_1(\epsilon t,\tau ) + O(\epsilon ^2) \nonumber \\ \frac{\vert \frac{\partial A_1}{\partial \epsilon t}\vert }{\vert \frac{\partial A_1}{\partial \tau }\vert }\sim & {} O(1) \end{aligned}$$

Dropping all terms apart from the very first and omitting the subscript 0 for notational convenience then yields Eq. (1).

In the remainder of this paper it is therefore implicitly assumed that the evolution with time *t* of $A(t,\tau )$ is slow, compared to the behaviour of $A(t,\tau )$ with $\tau$.

For what follows it is convenient to express Eq. (1) in terms of the cumulative number of infections:

8 $$\begin{aligned} C(t)=\int \limits _0^t j(t') \mathrm{d}t' \end{aligned}$$

It is straightforward to demonstrate by partial integration that Eq. (1) can be rewritten in terms of this cumulative number:

9 $$\begin{aligned} j(t)=-\left[ A(\tau )C(t-\tau )\right] +\int \limits _0^{\infty }A'(\tau )C(t-\tau ) \mathrm{d}\tau \end{aligned}$$

where $A'(\tau )$ is the first derivative of $A(\tau )$ with respect to $\tau$.It can be assumed that in the limit for $\tau \rightarrow \infty$ the function $A(\tau )$ vanishes and *C*(*t*) is bounded, so that the term in square brackets on the righthand side of (9) vanishes. The integral in Eq. (9) expresses what is known as a convolution of the functions $A'$ and *C*:

10 $$\begin{aligned} j(t)= & {} \int \limits _0^{\infty }A'(\tau )C(t-\tau ) \mathrm{d}\tau \nonumber \\= & {} \int \limits _{-\infty }^{\infty }A'(t-\widetilde{\tau })C(\widetilde{\tau }) \mathrm{d}\widetilde{\tau } \end{aligned}$$

where the second form, with $\widetilde{\tau }\equiv t-\tau$ is a more standard form. Adjusting the integration limit from 0 to $-\infty$ implies that it is assumed that for $\tau <0$ the functions $A'$ and *A* are identical to 0.

The problem of reconstructing $A'(\tau )$, and by extension $A(\tau )$, from a Fredholm-type equation such as (9), is an inverse problem for which many techniques exist. What is slightly more unusual is that in this case not only is the left-hand side *j*(*t*) measured data, but so is *C*(*t*). This type of problem is not unique to epidemiology. In astrophysics there is a mathematically very similar observational problem when reconstructing the distribution of gas clouds around the black holes at the centre of active galaxies (AGN) cf. Blandford and McKee (1982). Fluctuations in the continuum brightness of light sources very close to the black hole play the same role that *C*(*t*) does in the current setting, whereas the absorption and re-emission of light by certain spectral lines have the role of *j*(*t*). The equivalent of $A'(t)$ is called the transfer function in this field known as reverberation mapping of AGN. In reverberation mapping as well as here, the transfer function itself may also vary in time cf. Wanders (1995), albeit slowly compared to light travel times. In the case of AGN there are some additional problems such as the fact that it is impossible to obtain a perfectly regularly sampled time series. A method to deal with these problems, referred to as SOLA, is presented in Pijpers and Wanders (1994).

## Simple method

In the case at hand, where daily sampling is available, it is possible to make certain shortcuts in the method as compared to the implementation of the SOLA method Pijpers and Wanders (1994). Starting point is the integral Eq. (5) relating *j*(*t*) and *C*(*t*). These are both available as time series, sampled daily. In the case of the spread of covid-19 in the Netherlands the time series are still relatively short.

For what follows it is useful to recollect that time series analysis quite often makes use of Fourier transforms. A Fourier transform (FT) $F(\omega )$ of a time series is related to the original time series *f*(*t*) by:

11 $$\begin{aligned} F(\omega ) = \frac{1}{\sqrt{2\pi }} \int _{-\infty }^{\infty } f(t) e^{i\omega t} \mathrm{d}t \end{aligned}$$

in which $\omega$ is the frequency. The operation on *f*(*t*) is invertible, the inverse relationship is:

12 $$\begin{aligned} f(t) = \frac{1}{\sqrt{2\pi }} \int _{-\infty }^{\infty } F(\omega ) e^{-i\omega t} \mathrm{d}\omega \end{aligned}$$

In general, for an arbitrary real-valued function *f*, its FT is complex-valued. The operation is unique so that every integrable function and its FT can be referred to as an FT-pair. For discretely sampled time series, there are equivalent discrete versions (DFT) of these operations, with the same properties. There are a number of properties of Fourier transforms that are very useful in practice. Two of such proven theorems are of particular interest at the present. The first is that the FT of the derivative of a function is related to the FT of the function itself by:

13 $$\begin{aligned} FT(f')=-i\omega FT(\omega ) \end{aligned}$$

The second is that the FT of convolution integrals such as (10) can be performed very simply in the Fourier domain. The FT of a convolution of two functions is the product of the FT’s of those two functions. A common shorthand notation for a convolution operation is a $*$, so that:

14 $$\begin{aligned} FT(f*g)=FT(f)FT(g) \end{aligned}$$

When applying this theorem (14) to Eq. (10), The Result Is:

15 $$\begin{aligned} FT(j)=FT(A')FT(C) \end{aligned}$$

Making use of (13) then produces:

16 $$\begin{aligned} FT(j)=-i\omega \left[ FT(A)-\frac{K}{\omega ^2}\right] FT(C) \end{aligned}$$

The term with the constant *K* is necessary to introduce here. In setting up Eq. (1), or equivalently Eq. (10), only the endogenous spreading of infection is captured. The complementary exogenous process is where new infections come in to the system without have been caused by being infected by another individual within the population: for instance, from another spatial domain (another country) or by transfer from another species, e.g. animal-to-human infections. In Eq. (1) this would be represented by a Dirac delta-function term at $\tau =0$ : i.e. $K\delta (\tau )$ where *K* represents an average rate of inflow. In Eq. (1) with the cumulative numbers under the integral, this means that a term $K\tau$ must be subtracted from *A*, for which the Fourier transform is $K/\omega ^2$. This *K* is unknown but in practice is set by requiring the solution for $A=0$ for $\tau <0$. Naively one might therefore expect to be able to carry out either the mathematical operation:

17 $$\begin{aligned} A' = FT^{-1}\left( \frac{FT(j)}{FT(C)}-K\right) \end{aligned}$$

or the mathematical operation:

18 $$\begin{aligned} A = FT^{-1}\left( \frac{iFT(j)}{\omega FT(C)}+\frac{K}{\omega ^2}\right) \end{aligned}$$

This is problematic because for most time series, the Fourier transform can become 0 at some or even very many frequencies. If this happens to *FT*(*C*) it is clear that this leads to a division by 0 in Eqs. (17) and (18). In any case there is a problem with (18) at $\omega = 0$. This is one way to express the known fact that inverse problems are “ill-posed”. Another way to express this is that results of inversions are particularly sensitive to measurement errors in the data. The solution to this is to regularize the problem. There are a number of ways to achieve this regularization. Generally, the effect of regularization is that the result of the inversion is to produce a “smoothed” version of the function sought, i.e. $A'$ or *A* in this case. In other words, the data allows only a finite resolution in time for the reconstructed function.

For regularly sampled data there is a particularly straightforward way in which regularization can be achieved. To demonstrate this, consider again Eq. (15). Both left- and right-hand side of this equation can be multiplied by the complex conjugate of the FT of C:

19 $$\begin{aligned} FT(j)FT^{\dagger }(C) = FT(A') \vert FT(C)\vert ^2 \end{aligned}$$

where use is made of the fact that the product of a complex number or function with its complex conjugate is the, real-valued, square of the modulus. A regularized solution can now be obtained by taking:

20 $$\begin{aligned} A' = FT^{-1}\left( \frac{FT(j)FT^\dagger (C)-K\vert FT(C)\vert ^2}{\vert FT(C)\vert ^2+\mu FT_C^2(\omega =0)}\right) \end{aligned}$$

in which $FT_C(\omega =0)$ is the value of the FT of *C* at $\omega =0$, and $0< \mu <1$ is a weight parameter which acts as a “dial”to increase or decrease the extent of the regularization applied. To obtain *A* instead of $A'$ one would use:

21 $$\begin{aligned} A = FT^{-1}\left( \frac{i\omega FT(j)FT^\dagger (C)+K\vert FT(C)\vert ^2}{\omega ^2\vert FT(C)\vert ^2+\mu FT_C^2(\omega =0)}\right) \end{aligned}$$

Both Eqs. (20) and (21) are in effect applying Wiener filters to the data to regularize the inversion. Once *K* is determined for the determination of *A* using the procedure described in Sect. 4, the value can also be used to correct $A'$.

## Synthetic data

A usual procedure to test out methods for analysis of data, in particular where it concerns inverse methods, is to apply the method to synthetic data. In that case the true answer is known so that it becomes possible to compare the result of the data analysis with the truth. This is also a way to assess the influence of data errors. In principle, any model could be used to produce synthetic data. There are many models for epidemiological outbreaks, some highly sophisticated, see e.g. Grassly and Fraser (2008) or Liu et al. (2018) for an overview. For the present purpose it is sufficient to choose a few different analytical forms for the function $A(\tau )$ that are reasonably realistic to generate a time series and investigate the performance of the algorithm in reconstructing $A(\tau )$ from the time series.

- *Case 1 & 2 (Weibull)*
  22 $$\begin{aligned} A(\tau )=R_0 \frac{\beta }{\tau _m}\left( \frac{\tau }{\tau _m}\right) ^{\beta -1} e^{-\left( \frac{\tau }{\tau _m}\right) ^\beta } \end{aligned}$$
  For case 1 the parameter choices are $(R_0, \tau _m, \beta ) = (3, 7, 2)$ and for case 2 $(R_0, \tau _m, \beta ) = (3.745, 11, 0.8)$). This is sampled daily, i.e. for all integer values for $\tau$ from 1 to *T* inclusive. Outside of this range $A(\tau )$ is set to 0.
- *Case 3 (exponential)*
  23 $$\begin{aligned} A(\tau )= R_0 \frac{1}{\tau _m} e^{-\frac{\tau }{\tau _m}} \end{aligned}$$

Here $(R_0, \tau _m) = (4.73, 15.2)$. For all three cases $T=40$. This is then 0-padded for $\tau >T$ to a total length of the time series of 64. The time series for *j*(*t*) is generated iteratively by repeatedly convolving A and j to obtain the value of j for the next day, to a length of 40 days, using these two options for $A(\tau )$, with a starting value of 10 on day 1.

**Fig. 1:**
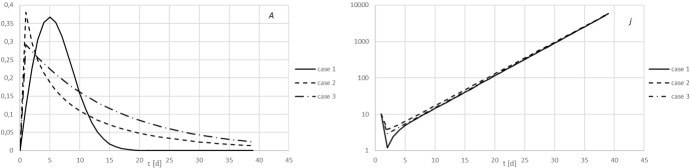
Left panel: the synthetic function $A(\tau )$ for cases 1 (solid line), 2 (dashed), and 3 (dash-dot). Right panel: the time series *j*(*t*) generated in case 1 (solid line), case 2 (dashed), and case 3 (dash-dot)

The function *A* and the resulting function *j* are shown in Fig. 1. The parameters are chosen deliberately to produce time series *j* that are very similar, even though the *A* that give rise to them are quite different. This demonstrates quite clearly the difficulty in reconstructing *A* from *j*. This lack of unicity is universal in solving inverse problems: there is a null-space of functions on the domain [0, *T*] which can be added to *A* without changing the integral (1). In general, the reproduction number that would be deduced by evaluating integral (6) will change when adding a function from the null-space to *A* so that even that is not well constrained. A further illustration of this issue can be found in Appendix1, which also demonstrates that this is a fundamental and unavoidable problem.

**Fig. 2:**
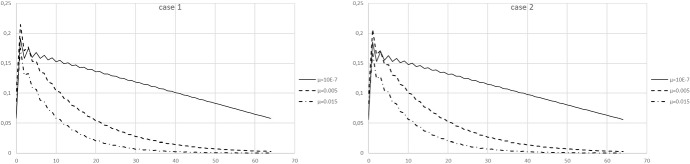
The result of the inversion of the data *j*(*t*) shown in Fig.1, for three different values of the regularization parameter $\mu$. Left panel is case 1, right panel is case 2

The results of applying the inverse method to the *j* time series for the cases 1 and 2 are shown in Fig. 2 for various choices of the regularisation parameter $\mu$. The results for cases 1 and 2 are barely distinguishable, and case 3 is so similar to the cases 1 and 2 shown here that it is omitted.

The exact same problem also occurs for any standard epidemiological modelling, i.e. a forward approach as opposed to an inverse approach: only if the precise form of *A* is already known a-priori from external considerations, will the data constrain the parameters of such a model. The time series *j* on its own, without additional knowledge, is insufficient to strongly constrain either *A* or the effective or basic reproduction numbers, regardless of whether one takes a forward/modelling approach or a non-parametric inverse approach.

To assess the influence of the length of the measured time series on the resulting inverted results, also a version of the case 1 is analysed where only the first 14 days of the time series is used rather than 40 days. The effect on the margins of uncertainty in particular is large: for the same values of the regularization parameter $\mu$ these errors increase by a factor of more than 10. At that level of uncertainty the resulting determination of *R* would still be acceptable, but the determination of the resolved transfer function *A* is no longer usable.

This conclusion does not automatically render modelling or inverse method approaches useless, however. The results from the inverse method presented here provide a “minimal solution” that is consistent with the data. The Wiener filtering that is applied (cf. Eqs. 20 and 21) will result in a solution for *A* that has the minimal structure or variation with $\tau$ that the data allows. Solutions for *A* which are valid but vary more as a function of $\tau$ than the minimal solution, may also have a different reproduction number, but will produce, by definition, the same time series for *j*. This is a distinct effect from uncertainties in the solution that are due to (administrative) data errors when recording that time series. The appendix outlines how the additional realisability constraint, that $A(\tau )\ge 0$ for all $\tau$, can be used to construct a range of allowed solutions and reproduction numbers. With this it becomes possible to disentangle the uncertainty in the reproduction number that arises from the inverse nature of the problem, from the uncertainty due to the influence of errors in the measured data.

## Publicly available covid-19 data

In principle, the best measurement would be if the time series for *j* and for *C* were known for the entire population. However, that would require either very extensive and repeated testing of the whole population, or at least regularly testing of (minimally) two independently obtained samples, representative of the population, and applying capture-recapture techniques to obtain statistical estimates for *j* and for *C* as a function of time. This approach is certainly very expensive as well as very labour-intensive and also not without risk. There might be risk to the medical staff who administer the tests for becoming infected, and also a risk that they then themselves potentially become a further source of infection for the populations that they test.

An alternative is therefore to apply this technique to a well-defined subset of the population. Ideally this would be an a-select sample, but at least a subset of the population that does not vary much in time in terms of its composition. A good candidate is the number of hospital admissions. The subset of the population that is infected and becomes sufficiently ill to need hospital care is probably the subset for which the registration is most timely and complete. This subset may well not be a-select but there is probably little change over time of the characteristics of the subset of the population which is most adversely affected.

To demonstrate this technique the publicly available hospitalisation data is used that is made available on the Dutch National Institute for Health and the Environment (RIVM) website. The update of April 2 is used, for which it is known that the most recent days in that dataset might not yet be complete. This is clearly the case for that date of April 2 itself. In order to make the most of the limited dataset, all of the other days are used, so any inaccuracies or incompleteness of the data will be reflected in the reconstructed $A'$ and *A*.

**Fig. 3:**
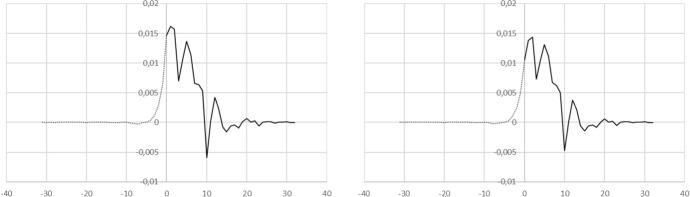
Determination of $A'$ using Eq. (20) and the daily hospitalisation numbers $j(t_i)$ and cumulative numbers $C(t_i)$ : (a. left panel) with $K=0$ , (b. right panel) adjusting $K\approx 0,00993$ to obtain $A\approx 0$ for $\tau <0$. The DFT yields results in wrap around order. The plotting is done in such a way that if $A'\ne 0$ at a positive $\tau$ , this implies that *j* is delayed with respect to *C* as would be expected. The dotted part of the curve should therefore be identical to 0, in the absence of regularization

**Fig. 4:**
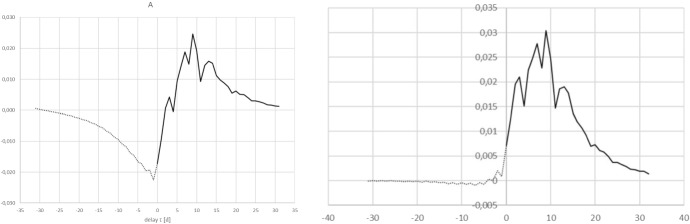
Determination of *A* using equation (21) with $K=0$ and the daily hospitalisation numbers $j(t_i)$ and cumulative numbers $C(t_i)$ : (a. left panel) with $K=0$ , (b. right panel) adjusting $K\approx 0,00993$ to obtain $A\approx 0$ for $\tau <0.$. The DFT yields results in wrap around order. The plotting is done in such a way that if $A\ne 0$ at a positive $\tau$, this implies that j is delayed with respect to C as would be expected

The FT is carried out using an FFT, which is a fast implementation of a DFT. This requires a length of the time series equal to $2^m$ where *m* can be any integer value. In this case $m=6$ and the measured time series is left padded with values of 0. The resulting time series $A(\tau )$ from the inverse DFT produces, in a wrap-around manner, the values for $A(\tau )$ for $\tau =-31,-30,\ldots ,0 ,1,\ldots ,32$. The result is plotted in Fig. 3 in the sense that if $A'\ne 0$ at a positive $\tau$, this implies that *j* is delayed with respect to *C* as would be expected. This is the black part of the curve. The dotted part of the curve is acausal and should therefore be $=0$. In practice however, there is the need to regularize in order to obtain a result that is not dominated by noise. The smoothing implied by the regularization means that some of the signal at $\tau >0$ “leaks” into the domain for $\tau <0$. The result shown uses $\mu =0.025$. The steep decline and negative value of $A'$ at 10d implies that *A* itself very likely declines sharply after 10 d. Note that all of the equations and therefore also all results are defined in terms of $\widetilde{\tau }$. In order to obtain $A'$ and *A* in terms of the original $\tau$ the signs must be reversed, or equivalently the complex conjugate taken before carrying out the inverse FT in resp. Eqs. (20) and (21). Determining *A* from Eq. (21) is straightforward numerically. If the unknown integration constant *K* is set to 0, the issue is that the value of the FT is set explicitly to 0 at $\omega =0$. In combination with the wrapping around and the regularization smoothing, the result of this is that at negative $\tau$ a broad negative “wing” is produced. One can also interpret this as being the integral of the (unphysical) dotted part of Fig. 4. An unfortunate side effect of this same smoothing is that then also for time delays of 1 and 2 days the value of $A(\tau )<0$. This is also unphysical. For this reason, the value of *K* must be determined by minimising E:

24 $$\begin{aligned} E=\int _{-\infty }^0 A^2(\tau ) \mathrm{d}\tau \end{aligned}$$

This is straightforward to carry out numerically, for instance using Brent’s method for which only function evaluations for successive estimates of K are necessary. In practice fewer than ten iterations provide sufficient precision. The result is shown in the right hand panels of Figs. 3 and 4 for $A'$ and *A* respectively.

It would appear that *A* peaks at around 10 days, after which there is a decay to around 20d, i.e. there is some remaining likelihood of secondary infection for about 3 to 4 weeks. A simple quadrature of *A* from day 0 up to day 32 (inclusive) yields a value of 0.41 which would be the best estimate of *R*(*t*) on that date. Note that this estimate uses all of the data available and is therefore in this sense an average of *R*(*t*) from the start of the outbreak up to April 2, corresponding to the integrating the lowest order term in the expansion shown in Eq. (7).

## Error propagation

It is known that inverse methods can be sensitive to data errors. For this reason, regularisation is always applied, implemented in the method discussed here through the parameter $\mu$. It is nevertheless important to actually quantify the margin of uncertainty on the results. If the method is linear, i.e. a linear combination of the measurement data, this is straightforward to carry out. In the present case, the propagation of measurement errors is not quite so simple, since measurement errors in *j* and in *C* are correlated. A further complication is that the character of the measurement errors is difficult to establish from the data themselves.

Therefore, while the reproduction number *R*(*t*) can be determined non-parametrically, in these circumstances it is inevitable that for the margin of uncertainty some parametrisation is needed at present. Two plausible parametrisations for the measurement error are presented here. The daily number of hospitalisations is treated as a Poisson process. For every day the expectation value, i.e. the value of the rate parameter $\lambda$, of that process is taken to be a moving average of the actually measured value on that day, together with the two previous and two subsequent days. For the days at the ends of the time series the value of $\lambda$ is kept constant.

- In the first variant, error model a, the measurement error is modelled by drawing random numbers satisfying such a Poisson process and perturbing the actually measured daily rates *j* using the difference between the random number drawn and the expectation value for that day. This is done 1000 times for every day in the series. The cumulative time series *C* is recalculated for every of the 1000 realisations for *j* so that the two are consistent. The entire inversion is repeated 1000 times to obtained propagated error margins.
- In the second variant, error model b, the same procedure is followed as described above. However, the parameter $\lambda$ is modified by assuming there are two contributing factors, so that: $\lambda =\lambda _{mov.av.}\left[ A_{mis}+(1-A_{mis})e^{(t_i-t_N)/\Delta }\right]$ The term $A_{mis}$ is a constant: it is assumed that this part of the measurement error is “misidentification”due to imperfect sensitivity and specificity of the tests for covid-19. For the simulations a value of 0.01 (i.e. 1% for the sum of false positives and negatives from tests) is assumed. The second term in this description expresses that there is a source of measurement error due to administrative delays in registration of admissions so that the data of the most recent few days are much more uncertain than the rest. A $\Delta =1.3$ is used, which implies the assumption that 90% of all administrative corrections are processed within 3 days.

**Fig. 5:**
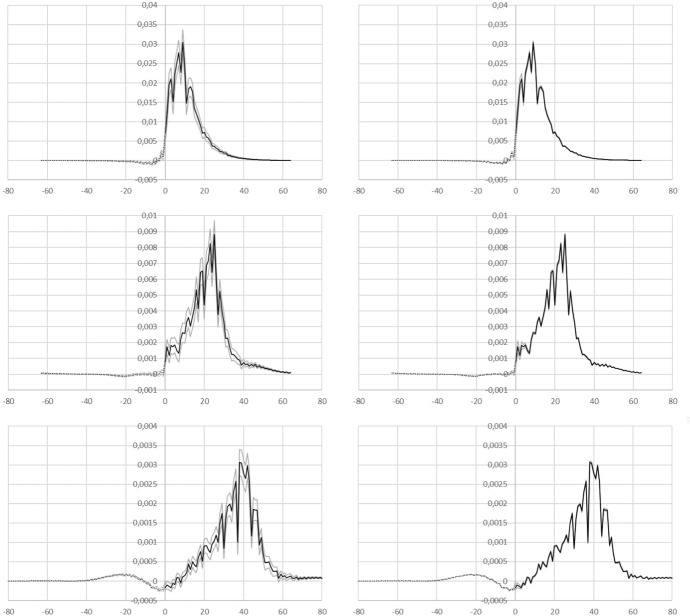
Left hand panels show $A(\tau )$ with uncertainty margins under error model a, right hand panels show error model b. In all panels the grey lines provide the margins of $\pm 2,58\sigma$ around $A(\tau )$. Top row: all data up to April 2, 2020. Middle row: all data up to April 18 2020. Bottom row: all data up to May 4 2020. Note that the scale of the abscissa changes between the rows of panels

The results of the error simulations are shown in Fig. 5, which shows the central result as a black line and the $\pm 2.58\sigma$ margins around this as grey lines. A broader zero padding is used for the data up to April 2 than in the previous figure to allow comparison with the data for April 18 in the middle row. The bottom row of panels shows the results when all data up to May 4 is included in the inversion. Clearly under error model b (the right column of panels), the uncertainty in the result is visible only for small values of $\tau$ whereas under model a (left column of panels), the margin is larger and can be seen for all $\tau$.

The determinations of *K* and of *R*(*t*) and the error estimates on these resulting from the two different assumptions regarding the measurement error are shown in Table 1. Comparing the top row and the middle row in Fig. 5, it can be seen that between April 3 and April 18 the peak near $\tau =10$ has moved along by the same number of days as the difference in the end-date of data collection: social distancing and other measures appear to be reflected in the suppression of A visible for small values of $\tau$.

**Table 1:** The values of *K* and of *R*(*t*) with $1\sigma$ error estimates under models a and b for the measurement errors, when including data only up to the three dates shown in the first column

Date	*K*	$\sigma (K)$ (a)	$\sigma (K)$ (b)	*R*(*t*)	$\sigma (R)$ (a)	$\sigma (R)$ (b)
02/04/2020	0.00993	0.0001	7 $\times 10^{-5}$	0.405	0.006	0.003
18/04/2020	0.00231	2 $\times 10^{-5}$	6 $\times 10^{-6}$	0.145	0.001	0.0004
04/05/2020	8.01 $\times 10^{-4}$	4 $\times 10^{-6}$	7 $\times 10^{-7}$	0.0609	0.0003	6 $\times 10^{-5}$

The effective reproduction rate *R*(*t*) is consistently dropping over the month of April. The (average) exogeneous fraction of infections *K* is also decreasing. Given the restrictions on foreign travel it is quite likely that most exogenous infections occurred only before the end of March. In principle this would mean that rather than a constant *K* it might be better to allow multiple terms in an expansion of a time-dependent $K(\tau )$ to the extent that these can be determined from the data. The bottom row of panels shows a wave structure at negative $\tau$ which might be due to neglecting the next order term in such an expansion. Further, it is of interest to note that in the panels in the bottom row, pronounced downward spikes are seen in particular at $\tau =$ 30d, 37d, and 44d. This is suggestive perhaps of a weekly modulation of hospital admissions being reflected in $A(\tau )$.

With these determinations of *A* for three different dates in hand, it is also possible to compare, at least crudely, the rates of change of *A* with $\tau$ and with *t* using simple first order finite differences. This is relevant because it allows checking whether the approximation (7) is at all justified. It appears that the rate of change of *A* with $\tau$ is on average a factor of roughly 3 to 4 larger than the rate of change with *t*. While this does imply that $\epsilon <1$ there is not as clear a separation of time scales as would be desirable to properly justify the approximation. The implication is that some of the change of *A* with time may feed into a change of *A* with $\tau$ so that, when *A* decreases with time as is the case here, the current determination of *A* and therefore also *R*(*t*) suffer from a downward bias of at most $\sim 25\%$ of their values.

## Left-truncating the time series

It is also of interest to determine what the effect can be of left-truncating the time series. If the assumption is allowed that the most recent cases of hospitalisation cannot have been caused in a direct link by people who have been hospitalised more than for instance 3 or 4 weeks ago, it might be reasonable to consider only the most recent 3 or 4 weeks, and remove all earlier data from the time series. For this reason, two variants are inverted. Variant a. is the time series of hospital admissions starting on April 22 and ending on May 20 (both dates inclusive), and therefore ignoring all previously recorded admissions. The second variant (b.) shortens this by a further week to include only the admissions between April 30 and May 20. For both variants a quite low value of the error weighting parameter $\mu =0.002$ is used, as well as a value $\mu _C=\sqrt{n_{\mathrm{days}}/\sum _{i=1}^{n_{\mathrm{days}}} C(t_i)}$ which can be argued to be a good scaling of the weighting needed. For variant a. this means $\mu =0.042$ , for variant b. it becomes $\mu =0.06$.

**Table 2:** The values of *K* and of *R*(*t*) with $1\sigma$ error estimates under models a and b for the measurement errors, when including data only up to the three dates shown in the first column

Date	$K\ \ \mu =0.002$	$K\ \ \mu =\mu _C$	$R\ \ \mu =0.002$	$R\ \ \mu =\mu _C$
22/04–20/05	$7.8 \times 10^{-4}\pm 2 \times 10^{-5}$	$2.37 \times 10^{-3}\pm 6 \times 10^{-5}$	0.375 ± 0.009	0.055 ± 0.001
30/04–20/05	$1.39 \times 10^{-3}\pm 6 \times 10^{-5}$	$4.5 \times 10^{-3}\pm 4 \times 10^{-4}$	0.64 ± 0.03	0.079 ± 0.003

One of the effects of the left-truncation must be that relatively more cases are considered to be from an external source, i.e. those hospitalisations that are near the beginning of the truncated series. This is indeed seen in Table 2, in that *K* increases when more of the actual series is cut off. Also, in the most recent weeks the numbers of hospitalisations are declining more and more gradually. For a perfectly constant number of new admissions, the $R(t)\equiv 1$, so it is not surprising that under these circumstances, shortening the time series makes *R*(*t*) increase to tend to 1. The differences in the values of *K* and *R*(*t*) for the two different values of error weighting $\mu$ are striking. The role of the error weighting for this particular inverse problem is not only to reduce the propagated data uncertainties. A larger $\mu$ also suppresses large amplitudes in $A(\tau )$ and hence reduces *R*(*t*). The smaller values for *R*(*t*) are consistent with the data, and arguably the results for the smaller values of $\mu$ are a result of too little regularisation, or over-fitting in the sense that the data do not sufficiently constrain *A* as described in section 33.

## Conclusions

This note demonstrates a non-parametric method to determine the effective reproduction number *R*(*t*) as the integral of the effective reproduction $A(t, \tau )$, and also to determine its first derivative with respect to $\tau$: $A'(t, \tau )$. It is applied to the hospital intake for the current covid-19 epidemic in the Netherlands. The approach is through solving an inverse problem, for which there are various techniques. The simplest technique is demonstrated, but this does not automatically produce good error estimates or confidence intervals for the functions $A(t, \tau )$ and $A'(t, \tau )$. Error estimates can be achieved by simulation but this requires assuming a model for the data error, with parameterisation, for the behaviour of the errors.

The current simple implementation of the method, using Fourier Transforms, is very fast indeed so that updates can easily be run real time.

By approaching the determination of the reproduction number as an inverse problem, it also becomes clear why any approach, including forward modelling approaches, will yield large margins of uncertainty. These margins are not primarily a consequence of errors or uncertainty in the measured data, but instead are a consequence of the nature of the mathematical problem.

In the appendix to this paper it is demonstrated that time series data do not constrain the form of the transfer function *A* very well, and by extension also do not provide strong constraints on *R*(*t*) without further assumptions or a-priori knowledge. It is argued that larger estimates of *R*(*t*) may be caused by overfitting / under-regularising the models. In essence any finite amount of time series data allows a large (infinite) null-space of solutions for the transfer function which can be added at will but have a non-zero contribution to both *A* and *R*. Conversely, if the larger values for *R*(*t*) reported elsewhere are correct, considering a combination of virology and behavioural considerations, the time series data add relatively little to the evidence for this. One should not conclude from this that the non-parametric method cannot be gainfully applied here. Rather, the inversion produces an objective reference result, and therefore illuminates which aspects of the modelling of the transfer function *A* require justifications that are external to these measured data.

The error propagated from random administrative and measurement errors is relatively modest compared to these unicity problems. However, experiments using synthetic data demonstrate that time series as short as two weeks are so short that then the random error becomes unacceptably large; increasing in the synthetic example by a factor of 10 when the time series is shortened from 6 weeks to 2 weeks. This means that in the very earliest stages of an epidemic the time series are probably too short to produce confidence intervals for the transmission function *A* that are small enough for the result to be meaningful. If control measures, intended to suppress *A*, change often over the typical time scales of transmission, the quality of the determination of $A(t,\tau )$ and by extension *R*(*t*) is adversely affected, due to the mixing of the dependence of *A* on *t* and $\tau$.

## Appendix: null-spaces in idealised cases, and non-uniqueness of A

As an illustration of the influence of the null-space in the solutions of Eq. (1) it is useful to consider a few special cases for *j*. The first step is to consider a given *j*(*t*) and hypothesize that two different $A(\tau )$ could have given rise to that same *j*:

25 $$\begin{aligned} j(t)= & {} \int \limits _0^{\infty } A_1(\tau ) j (t-\tau )\mathrm{d}\tau \nonumber \\ j(t)= & {} \int \limits _0^{\infty } A_2(\tau ) j (t-\tau )\mathrm{d}\tau \end{aligned}$$

Subtracting these two produces:

26 $$\begin{aligned} 0= & {} \int \limits _0^{\infty } \left[ A_2(\tau )-A_1(\tau )\right] j (t-\tau )\mathrm{d}\tau \nonumber \\\equiv & {} \int \limits _0^{\infty } B(\tau ) j (t-\tau )\mathrm{d}\tau \end{aligned}$$

It is straightforward to demonstrate that there are non-trivial solutions for $B(\tau )$ for any given *j*, i.e. solutions other than $B(\tau )=0$.

*Exponentially increasing* *j* As a first example, consider a purely exponentially increasing *j*, so that solutions are sought for:

27 $$\begin{aligned} 0= & {} \int \limits _0^{\infty } B(\tau ) j_0 e^{\alpha (t-\tau )}\mathrm{d}\tau \nonumber \\= & {} \frac{j_0}{\alpha }e^{\alpha t} \int \limits _0^{\infty } B\left( \frac{x}{\alpha }\right) e^{-x} \mathrm{d}x \end{aligned}$$

with $x\equiv \alpha \tau$. The factor outside the integral sign is not $=0$ and can therefore be ignored. There is a set of orthogonal polynomials, known as Laguerre functions, with notation $L_n (x)$ defined for all $n=0,1,2,\ldots$ for which the orthogonality condition holds that:

28 $$\begin{aligned} \int \limits _0^{\infty } L_n(x) L_m(x) e^{-x} \mathrm{d}x = \delta _{nm} \end{aligned}$$

in which $\delta _{nm}$ is the Kronecker delta (i.e. $=1$ if $n=m$, and $=0$ otherwise). Explicit expressions for $L_n (x)$ are:

29 $$\begin{aligned} L_n(x) = \sum \limits _{k=0}^{n} (-1)^k\begin{pmatrix} n\\ n-k \end{pmatrix}\frac{1}{k!}x^k \end{aligned}$$

For $n=0$ Eq. (29) produces $L_0 (x)=1$. Using this in combination with the orthogonality condition (28) implies that:

30 $$\begin{aligned} \int \limits _0^{\infty } L_n(x) e^{-x} \mathrm{d}x = \delta _{n0} \end{aligned}$$

which means that the integral in (30) is $=0$ for all $n\ge 1$. This means that a function *B* defined as

31 $$\begin{aligned} B(\tau )= \sum \limits _{n=1}^{\infty } b_n L_n(\alpha \tau ) \end{aligned}$$

will always satisfy equation (27), where every of the constant coefficients $b_n$, i.e. an infinite number of free parameters, can be chosen completely at will. This implies that once any solution $A(\tau )$ is found so that (25) is satisfied, an infinite number of alternative solutions can be constructed by adding any function of the form (31).

*Polynomially increasing* *j* As a second example, consider instead a polynomially increasing function $j(t)=j_0 t^\alpha$ , with $\alpha >0$ over a domain from $t=0$ to $t=T$, so that solutions are sought for:

32 $$\begin{aligned} 0= & {} \int \limits _0^{t} B(\tau ) j_0 (t-\tau )^{\alpha }\mathrm{d}\tau \nonumber \\= & {} j_0\left( \frac{t}{2}\right) ^{\alpha +1} \int \limits _{-1}^{1} B\left( (x+1)\frac{t}{2}\right) (1-x)^{\alpha } \mathrm{d}x \end{aligned}$$

with $x\equiv \frac{2\tau }{t}-1$. The factor outside the integral sign again is not =0 and can therefore be ignored. There is a set of orthogonal polynomials, known as Jacobi functions, with notation $P_n^{(\alpha ,0)}(x)$ defined for all $n=0,1,2,\ldots$ for which the orthogonality condition holds that:

33 $$\begin{aligned} \int \limits _{-1}^{1} P_n^{(\alpha ,0)}(x) P_m^{(\alpha ,0)}(x) (1-x)^{\alpha } \mathrm{d}x = \frac{2^{\alpha +1}}{2n+\alpha +1}\delta _{nm} \end{aligned}$$

Explicit expressions for $P_n^{(\alpha ,0)} (x)$ are:

34 $$\begin{aligned} P_n^{(\alpha ,0)}(x) = \frac{1}{2^n}\sum \limits _{k=0}^{n} (-1)^k\begin{pmatrix} n+\alpha \\ k \end{pmatrix}\begin{pmatrix} n\\ n-k \end{pmatrix}(x-1)^{n-k}(x+1)^k \end{aligned}$$

For $n=0$ equation (34) produces $P_0^{(\alpha ,0)}(x)=1$. Using this in combination with the orthogonality condition (33) implies that:

35 $$\begin{aligned} \int \limits _{-1}^{1} P_n^{(\alpha ,0)}(x) (1-x)^{\alpha } \mathrm{d}x = \frac{2^{\alpha +1}}{2n+\alpha +1}\delta _{n0} \end{aligned}$$

This means that a function *B* defined as:

36 $$\begin{aligned} B(\tau )= \sum \limits _{n=1}^{\infty } b_n P_n^{(\alpha ,0)}\left( \frac{2\tau }{t}-1\right) \end{aligned}$$

will always satisfy Eq. (32)), where once again every of the constant coefficients $b_n$, i.e. an infinite number of free parameters, can be chosen completely at will. This implies that once any solution $A(\tau )$ is found so that (25) is satisfied, an infinite number of alternative solutions can be constructed by adding any function of the form (36).

*A **j**increasing and decreasing, following a bell curve* A third example is for a j satisfying:

37 $$\begin{aligned} j(t)= j_m e^{-\left( \frac{t-t_m}{\Delta }\right) ^2} \end{aligned}$$

In this case solutions are sought for:

38 $$\begin{aligned} 0= & {} \int \limits _0^{\infty } B(\tau ) j(t-\tau )\mathrm{d}\tau \nonumber \\= & {} j_m\Delta \left( \frac{t}{2}\right) ^{\alpha +1} \int \limits _{-\infty }^{\infty } B\left( t-t_m-x\Delta \right) e^{-x^2}\mathrm{d}x \end{aligned}$$

with $x\equiv (t-t_m-\tau )/\Delta$.Note that the lower limit of the integration is adjusted, which is allowed as long as $\Delta$ is small enough, compared to $\vert t-t_m \vert$. There is a set of orthogonal polynomials, known as Hermite functions, with notation $H_n (x)$ defined for all $n=0,1,2,\ldots$ for which the orthogonality condition holds that:

39 $$\begin{aligned} \int \limits _{-\infty }^{\infty } H_n(x) H_m(x) e^{-x^2} \mathrm{d}x = 2^n n!\sqrt{\pi }\delta _{nm} \end{aligned}$$

Explicit expressions for $H_n$ are:

40 $$\begin{aligned} H_n(x) = n!\sum \limits _{k=0}^{n/2} (-1)^k \frac{1}{k!(n-2k)!} (2x)^{n-2k} \end{aligned}$$

For $n=0$ Eq. (40) produces $H_0 (x)=1$. Using this in combination with the orthogonality condition (39) implies that:

41 $$\begin{aligned} \int \limits _{-\infty }^{\infty } H_n(x) e^{-x^2} \mathrm{d}x = 2^n n!\sqrt{\pi }\delta _{n0} \end{aligned}$$

This means that a function *B* defined as:

42 $$\begin{aligned} B(\tau )= \sum \limits _{n=1}^{\infty } b_n H_n\left( \frac{t-t_m-\tau }{\Delta }\right) \end{aligned}$$

will always satisfy Eq. (38), where once again every of the constant coefficients $b_n$, i.e. an infinite number of free parameters, can be chosen completely at will.

*A* *j* *increasing and decreasing asymmetrically, following long-tailed polynomial behaviour* A final example is for a *j* satisfying:

43 $$\begin{aligned} j(t)=2j_m\frac{\left( t/t_m\right) ^{\frac{\beta }{2}-1}}{1+\left( t/t_m\right) ^{\beta }} \end{aligned}$$

which, while not a formal fit to the data for the Netherlands, has a reasonably similar shape, for $(t_m,j_m,\beta )=(33,540,8.8)$. In this case, solutions are sought for:

44 $$\begin{aligned} 0= & {} \int \limits _0^{\infty } B(\tau ) j(t-\tau )\mathrm{d}\tau \nonumber \\= & {} 2j_mt_m \int \limits _{0}^{t/t_m} B\left( t-x t_m\right) \frac{x^{\frac{\beta }{2}-1}}{1+x^\beta }\mathrm{d}x \end{aligned}$$

where adjusting the upper limit of the integration is allowed, assuming that $j=0$ for $t<0$. Here $x\equiv (t-\tau )/t_m$. In this case it is convenient to use a further change of variable. Define:

45 $$\begin{aligned} \phi \equiv \frac{1}{r}\arctan \left( x^{\frac{\beta }{2}}\right) -1 \end{aligned}$$

in which:

46 $$\begin{aligned} r\equiv \frac{1}{2}\arctan \left( \left( \frac{t}{t_m}\right) ^{\frac{\beta }{2}}\right) \end{aligned}$$

Using these definitions, Eq. (44) can be rewritten as:

47 $$\begin{aligned} 0= 2j_m t_m\int \limits _{-1}^{1} B\left( t -t_m\left( \tan \left( r(\phi +1)\right) \right) ^{\frac{2}{\beta }}\right) \mathrm{d}\phi \end{aligned}$$

There is a set of orthogonal polynomials, known as Legendre functions, with notation $P_n (x)$ defined for all $n=0,1,2,\ldots$ for which the orthogonality condition holds that:

48 $$\begin{aligned} \int \limits _{-\infty }^{\infty } P_n(\phi ) P_m(\phi ) \mathrm{d}\phi = \frac{2}{2n+1}\delta _{nm} \end{aligned}$$

Explicit expressions for $P_n$ are:

49 $$\begin{aligned} P_n(x) = \frac{1}{2^n}\sum \limits _{k=0}^{n/2} (-1)^k \begin{pmatrix}n\\ k\end{pmatrix}\begin{pmatrix}2n-2k\\ n\end{pmatrix} \phi ^{n-2k} \end{aligned}$$

For $n=0$ Eq. (49) produces $P_0 (\phi )=1$. Using this in combination with the orthogonality condition (48) implies that:

50 $$\begin{aligned} \int \limits _{-\infty }^{\infty } P_n(\phi ) \mathrm{d}\phi = \frac{2}{2n+1}\delta _{n0} \end{aligned}$$

This implies that a function *B* defined as:

51 $$\begin{aligned} B(\tau )=\sum \limits _{n=1}^{\infty } b_n P_n\left( 2\frac{\arctan \left( \left( \frac{t-\tau }{t_m}\right) ^{\frac{\beta }{2}}\right) }{\arctan \left( \left( \frac{t}{t_m}\right) ^{\frac{\beta }{2}}\right) }-1\right) \end{aligned}$$

will always satisfy Eq. (44), where once again every of the constant coefficients $b_n$, i.e. an infinite number of free parameters, can be chosen completely at will.

These four examples illustrate a more general principle that for any positive semi-definite weight function *w* a set of polynomials $F_n$ over a (possibly infinite) interval (*a*, *b*) can be defined, satisfying an orthogonality condition:

52 $$\begin{aligned} \int \limits _a^b F_n(x)F_m(x) w(x)\mathrm{d}x = \delta _{nm} \end{aligned}$$

In the present application the measured function *j*, which is positive semi-definite, plays the role of the weight function *w*. For a general *j* such functions are not likely to be named and known orthogonal functions, but nevertheless series expansions of such polynomials can always be constructed recursively. The implication is that for any measured *j*, as soon as one valid solution *A* is constructed, a whole family of solutions ${\widetilde{A}}$ can be found that are all consistent with the data and that are therefore not constrained by the data:

53 $$\begin{aligned} {\widetilde{A}}(\tau ) \equiv A(\tau )+B(\tau )\equiv A(\tau )+\sum _{n=1}^{\infty } b_nF_n(\tau ) \end{aligned}$$

There is another constraint on *A*, however, which is that $A(\tau ) \ge 0$ for all $\tau$. Not every possible set of coefficients $\{b_n \}$ will produce an alternative solution $A(\tau )$ that satisfies that constraint, but there is always sufficient freedom to construct solutions that do.

This result also has the implication that the basic reproduction number $R_0$ or effective reproduction number *R*(*t*) are only poorly constrained by just the measured *j*(*t*). While the construction of the orthogonal functions implies that:

54 $$\begin{aligned} \int \limits _a^b \left[ A(\tau )+B(\tau )\right] j(t-\tau ) \mathrm{d}\tau = j(t-\tau ) \end{aligned}$$

the reproduction number for those alternative solutions can be different however, i.e.:

55 $$\begin{aligned} \int \limits _a^b \left[ A(\tau )+B(\tau )\right] \mathrm{d}\tau \ne \int \limits _a^b A(\tau ) \mathrm{d}\tau \end{aligned}$$

Additional information beyond a measured time series *j* is therefore needed to enable constraining the reproduction number.
